# Dual Mechanism of Ion Permeation through VDAC Revealed with Inorganic Phosphate Ions and Phosphate Metabolites

**DOI:** 10.1371/journal.pone.0121746

**Published:** 2015-04-10

**Authors:** Eva-Maria Krammer, Giang Thi Vu, Fabrice Homblé, Martine Prévost

**Affiliations:** Structure et Fonction des Membranes Biologiques, Centre de Biologie Structurale et de Bioinformatique, Université Libre de Bruxelles (ULB), Brussels, Belgium; Russian Academy of Sciences, Institute for Biological Instrumentation, RUSSIAN FEDERATION

## Abstract

In the exchange of metabolites and ions between the mitochondrion and the cytosol, the voltage-dependent anion channel (VDAC) is a key element, as it forms the major transport pathway for these compounds through the mitochondrial outer membrane. Numerous experimental studies have promoted the idea that VDAC acts as a regulator of essential mitochondrial functions. In this study, using a combination of molecular dynamics simulations, free-energy calculations, and electrophysiological measurements, we investigated the transport of ions through VDAC, with a focus on phosphate ions and metabolites. We showed that selectivity of VDAC towards small anions including monovalent phosphates arises from short-lived interactions with positively charged residues scattered throughout the pore. In dramatic contrast, permeation of divalent phosphate ions and phosphate metabolites (AMP and ATP) involves binding sites along a specific translocation pathway. This permeation mechanism offers an explanation for the decrease in VDAC conductance measured in the presence of ATP or AMP at physiological salt concentration. The binding sites occur at similar locations for the divalent phosphate ions, AMP and ATP, and contain identical basic residues. ATP features a marked affinity for a central region of the pore lined by two lysines and one arginine of the N-terminal helix. This cluster of residues together with a few other basic amino acids forms a “charged brush” which facilitates the passage of the anionic metabolites through the pore. All of this reveals that VDAC controls the transport of the inorganic phosphates and phosphate metabolites studied here through two different mechanisms.

## Introduction

ATP synthesis occurs in the matrix space of mitochondria. Its production is highly dependent on a rich supply of metabolites that are transported through both the inner and the outer membrane of the mitochondrion. Whereas metabolites are translocated through the inner mitochondrial membrane by specific protein translocases, the voltage-dependent anion channel (VDAC) forms the major transport pathway for ATP/ADP exchange and for the fluxes of other metabolites, cofactors, and inorganic ions through the outer mitochondrial membrane. The measured fluxes of ATP through VDAC are reported to be sufficient to provide all the ATP needed for the vital cell functions [1,2].

All organisms possessing mitochondria have at least one VDAC isoform whose fundamental properties, including single-channel conductance, selectivity, voltage dependence, and a positively charged lumen, are highly conserved. The mouse and human VDAC isoform 1 (mVDAC1 and hVDAC1) atomic level 3D structures were determined using either NMR, X-ray crystallography, or a combination of both methods [3–5]. More recently the structure of the zebrafish VDAC isoform 2 has been solved [6]. All these structures show a large β-barrel-shaped pore made of 19 β-strands and one N-terminal helical segment folded into the pore lumen. This N-terminal segment, however, adopts significantly different positions in the NMR structure of hVDAC1 [4], where it extends roughly through the middle of the pore, whereas in the other VDAC1 and VDAC2 structures, it adopts a helix aligned almost parallel to the plane of the membrane, creating a constriction in the central region of the pore. The latter position and orientation of the helix has been corroborated in a solid-state NMR study [7]. Although the biological significance of these 3D structures has been debated because of divergences with previously proposed empirical models [8,9], an increasing number of recent reports support their physiological relevance [10–17].

VDAC is proposed to regulate the transport of anionic metabolites, mediated by binding sites along its pore wall [18–20], via a gating process promoting the transition from an open channel to a closed state [1,2,21] or through the interaction of VDAC with different cytosolic proteins [22,23]. VDAC is thus thought to act as a global regulator of mitochondrial outer membrane permeability and hence of mitochondrial functions [10,24–26].

Fluxes of phosphates, nucleotides, and carboxylic acid metabolites through the VDAC open pore have been measured in numerous experimental studies [2,18,27–33]. NADH has been shown to regulate the permeability of mammalian, fungal, and plant VDACs to ADP [33] and also to control VDAC gating [32]. Because NADH interacts with VDAC and because VDAC sequence motifs feature similarities to those of well-known nucleotide-binding protein sequences, the existence of ATP binding sites inside the VDAC pore has been proposed [32]. Furthermore, an analysis of the ion current noise induced by different nucleotides has suggested that their permeation characteristics differ [31]. This study, performed at high NaCl concentration, suggested that NADPH, NADH, and ATP translocate through the pore via a specific binding mechanism, whereas UTP, AMP, and NAD are transported via a random walk process. Characterization of VDAC nucleotide binding sites with a photoactive ATP analog has indicated three possible sites, including one at the N-terminus and one at the C-terminus of VDAC [18]. The results of a site-directed mutagenesis study further support the existence of one N-terminal nucleotide binding site and point to a role for a lysine residue in this region [19]. In one NMR study, chemical shift changes were observed in human VDAC1 in the presence of β-NADH, but the study showed no specific binding site for ATP [4]. In contrast to these findings, the results of a recent NMR study suggest the existence of a fairly large binding region common to ATP, GTP, and UTP, which partially overlaps with a previously observed NADH binding location [20]. In agreement with these data the recently determined ATP-mVDAC1 complex structure has highlighted the occurrence of one ATP molecule loosely bound in the pore of mVDAC1 to basic residues on the N-terminal helix [34]. Altogether, these reports suggest that the mechanism of metabolite translocation through the pore of VDAC and the binding sites involved might depend on the type of phosphate metabolite to be transported. The detailed mechanism through which VDAC regulates the crossing of metabolites and how it compares to that of small inorganic ions remains to be understood.

The 3D structures determined so far are believed to represent the ‘open’ state of the channel and pave the way for the investigation of VDAC functional principles using atom-based simulation techniques. Previous reports have shown that though still challenging, these computational approaches can be used to study both ion translocation and selectivity of biological channels [35–37]. In particular, the transport of small inorganic ions through mVDAC1 and hVDAC1 has been extensively studied using molecular simulations and continuum electrostatics calculations [38–45]. Molecular dynamics (MD) and Brownian dynamics (BD) studies on mVDAC1 showed that the permeation of potassium chloride occurs without following specific pathways and without forming long lasting interactions with residues of VDAC pore [38–40]. Two recent MD studies undertaken on the VDAC-ATP complex system have shown the occurrence of low-affinity binding sites for ATP along its translocation pathway through VDAC [34,46]. These reports suggest that this metabolite permeates VDAC in a mechanistic way differing from small inorganic anions.

In the present study we address the issue of the existence of different permeation mechanism for anionic metabolites and inorganic anions. To this end we have combined classical and adaptive biasing force (ABF) MD simulations with electrophysiology measurements to examine the permeation of two anionic metabolites (ATP and AMP) and of the inorganic phosphate (P_i_) in two different protonation states. The ATP metabolite was chosen for its tremendous importance in the essential functions of the cell. AMP was investigated as its translocation features were suggested to differ from those of ATP [18,30]. The permeation of ATP and AMP was studied in two different KCl concentrations: one close to physiological conditions and the second at high salt concentrations, the latter condition having been used in a large number of experimental studies.

Our 6-μs simulation data showed that anions such as chloride and monovalent P_i_ (H_2_PO_4_^-^) experienced a similar translocation process featuring no specific pathways and no binding sites. In contrast the translocation mechanism proved dramatically different for the divalent form of P_i_ (HPO_4_^2-^) and for AMP and ATP, with the establishment of a specific permeation pathway connecting binding sites. The energetics of ATP and AMP permeation are also consistent with their effect on VDAC conductance, as measured in this study by electrophysiology. Overall our study highlights that VDAC exploits two different mechanisms to permeate either monovalent or higher valent anion species.

## Materials and Methods

All MD simulations were performed with the program namd2.9 [47]. The all-atom CHARMM27 force field [48,49] with CMAP corrections [50] was used for protein, water, and ions. The lipid molecules were described by a united atom force field [51]. All other parameter settings were defined as described elsewhere [38,40].

### Metabolite translocation assays

Five different randomly chosen locations of AMP and ATP on the cytosolic side (z >0) and on the intermembrane space side (z <0) of the channel were used initially to simulate their translocation through mVDAC1 (S1A Fig.). The cytosolic and intermembrane space sides of the protein were defined as in *Tomasello et al*. [52]. A pre-existing equilibrated MD system of mVDAC1 embedded in POPE was used as a starting point [40]. Two KCl concentrations were used: 0.1 M that represents the physiological salt concentration and 1 M that has been used in several experimental studies. For each system, an equilibration was carried out with the position of ATP or AMP kept fixed. After the release of the metabolite, simulations were performed in the absence and presence of a transmembrane potential (S1 Table). Those performed in the absence of transmembrane potential were discontinued after the metabolite was observed to remain close to the same protein residues for at least 2 ns. They lasted from 10 to 30 ns. Trajectories obtained in the presence of an applied transmembrane potential of 50 mV or 500 mV were produced for 50 ns. The transmembrane potential was imposed via an applied uniform electric field directed normally with respect to the lipid bilayer [53–56]. All simulations at nonzero external field were carried out in the NVT ensemble. Only in the simulations with an imposed voltage, the Cα atom positions were restrained using an harmonic force with a constant of 1 kcal/mol^.^Å^2^ [57].

The 10 simulations performed with the Mg^2+^-coordinated ATP started using the pre-equilibrated Mg-free ATP systems in which the position of a potassium ion bound to the γ- or β-phosphate group of ATP was replaced by a magnesium ion. We then carried out a 2.5 ns equilibration of the water and ions. In all 10 simulations the magnesium was observed to fit tightly between the γ- and β-phosphate groups of ATP as in a low-energy configuration observed for ATP in solution [58,59]. Trajectories in the presence of a 500-mV transmembrane potential were then produced for 50 ns as described for the ATP free form simulations.

### Free energy profile of metabolite permeation

The free energy landscape of AMP and ATP permeation was determined at 0.1 M and 1 M KCl using the ABF method [60,61] along the reaction coordinate z. The latter corresponded to the distance between the phosphorus atom of the terminal phosphate group and the geometric center computed from backbone carbon atom positions of the following protein residues, R15, V17, L58, F99, A141, S193, and L259, located at the center of the pore. A full translocation was considered when the metabolite travelled from -22 to 22 Å through the pore. This distance was divided into windows with a thickness ranging from 2 to 5 Å. To ensure a correct sampling of the metabolite both in each window space and in its internal conformations, several starting positions were extracted from the MD simulations performed with an applied transmembrane potential of 500 mV (for example, S1B Fig.). Additional sampling of about 60 ns was performed over the entire reaction pathway using two to three different starting positions of the metabolite along the reaction pathway. For the estimation of the error the overall reaction pathway was again split in 8 windows with a 5 to 7 Å thickness. In each window 10-ns trajectories were generated using two different starting positions for the metabolite molecule. The error estimation was performed as described in [60]. The overall simulation times were 940 ns, 985 ns, and 925 ns for the 0.1 M KCl ATP, 1 M KCl ATP, and 0.1 M KCl AMP profiles, respectively.

### Generation of the force field parameters for the phosphate ions

The force field parameters for H_2_PO_4_^-^ and HPO_4_^2-^ were produced using the program CGenFF with standard parameters [62]. The penalty values obtained for H_2_PO_4_^-^ and HPO_4_^2-^ were close to 10, thus below the recommended values for further optimization.

### Small ion permeation simulations

Different setups were generated for simulating 0.2 M NaCl, NaH_2_PO_4_, and Na_2_HPO_4_ permeation. Preexisting equilibrated MD systems of mVDAC1 embedded in POPE at 0.1 M KCl were used as starting points [40]. For each system, a different random distribution of the ions was generated. A 10-ns equilibration was performed, in which the protein atom positions were kept fixed. Two 100-ns simulations were generated as production runs, except for Na_2_HPO_4_, for which two additional 50 ns simulations were performed.

### Free energy profiles of inorganic ion permeation

The multi-ion free energy profile along the pore axis was calculated using the 0.2 M NaCl, NaH_2_PO_4_, and Na_2_HPO_4_ MD trajectories. The free energy of an ion species *i* inside the channel at position *z* is given by [63]:

$$\Delta G_{i}(z)=-RT\ln\frac{C_{i}(z)}{C_{bulk}},$$

where R, T, C_i_(z), and C_bulk_ are the gas constant, temperature, concentration inside the pore at z, and bulk concentration of the ionic species *i*, respectively. This relationship has been used to compute the free energy profiles of small compounds across different protein pores [64,65] and also of KCl inside VDAC [38,40,42].

### MD analysis

All trajectories were monitored for several types of interactions using vmd [66] and eucb [67]. A translocation event was defined as the event of an ion traveling across the pore axis from z<15 to z>-15 Å or vice versa. An electrostatic interaction between phosphate and protein residues was considered formed when the distance between at least one phosphate oxygen and a side chain nitrogen of a Lys or Arg residue was 4 Å or less. An interaction between phosphate and a cation was counted when the distance between the phosphate oxygen atoms and the cation was smaller than 4 Å. π-π interactions were defined to occur using the eucb program [67] (modified for accounting for AMP and ATP) when the distance of the center of mass of three atoms of the adenine ring to that of three atoms of Phe/Tyr/Trp ring was smaller than 6 Å and the angle between the two planes was lower than 30°. Cation-π interactions between the adenine moiety and basic residues were considered established when the distance from the center of mass of three atoms of the adenine ring to at least one nitrogen of the Lys/Arg side chain was smaller than 6 Å and the angle between the adenine plane and one of the NH vectors of the residue side chain was larger than 60°. The stacking of the Arg guanidinium moiety on top of adenine ring was considered using the same distance criterion but an angle value smaller than 30°. Hydrogen bonds between adenine ring nitrogen atoms and side chain oxygen atoms of protein residues were counted when the distance between the heavy atoms was less than 3.5 Å and the angle for the donor-hydrogen-acceptor was larger than 120°.

The positions of ions inside the pore were determined using the position of the terminal phosphorus atom for ATP and AMP. The time-averaged number of interactions along the pore axis was estimated by dividing the pore into several slices and calculating the number of snapshots featuring the interacting group divided by the total number of snapshots with the group in this slice. The thickness of the slice was defined as 1 Å for hydrogen bond, and electrostatic interactions and 2 Å for cation-π and π-π interactions.

### Electrophysiology experiments

The purified 32-kDa bean VDAC (PcVDAC) was reconstituted in planar lipid bilayers as described previously [68]. The bilayer membrane was formed from a 2% solution of soybean phospholipid extract (Avanti Polar Lipid, Alabaster, AL) in hexane by folding two lipid monolayers over a hole (130–140 μm in diameter) made in a 25-μm-thick Teflon sheet separating two Teflon experimental chambers. Before each experiment, the partition was treated with a solution of hexadecane/hexane (2.5%, v/v). Ag/AgCl electrodes connected in series with a salt bridge (1 M NaCl in 1% agar) were used to connect the experimental chambers to the electronic equipment. The *trans* compartment is defined as the one connected to the ground, and the voltage was applied to the *cis* compartment. For channel reconstitution into a planar lipid bilayer, proteins were added to the *cis* compartment. All solutions were buffered with 10 mM HEPES-NaOH at pH 7.5. Current recordings were performed as described previously [69], using a BLM 120 amplifier (BioLogic, France). Signals were filtered at 1 kHz (5-poles linearized Tchebichev filter).

Channel reconstitution was achieved in the presence of an identical NaCl concentration on both sides of the membrane. The *cis* compartment was afterwards perfused with three times its volume of a solution of different composition.

Membrane conductance was calculated from the current-voltage (I/V) curve recorded between +20 mV and -20mV. The I/V curve was first measured with an identical salt concentration on both sides of the membrane. Then, the *cis* compartment was perfused with three times its volume of the same NaCl solution supplemented with either AMP or ATP at 25 mM concentration, and a new I/V curve was recorded. As the presence of nucleotides increased the Na^+^ concentration in the *cis* compartment, it was necessary to probe the effect of a change in ion concentration on VDAC conductance. This was done by perfusing the *cis* compartment with 0.15 or 0.2 M NaCl, to mimic the effect of an excess of Na^+^ associated with AMP or ATP, respectively.

## Results

With the aim of investigating the possible occurrence of different permeation mechanisms through VDAC we examined the translocation of diverse anions: two anionic metabolites (ATP and AMP) and the phosphate ion (P_i_) in two different protonation states and we compared their permeation features to those of the Cl^-^ ion [38–40].

### Metabolite translocation

To study ATP and AMP permeation through VDAC, we first carried out 10 individual MD simulations of one single nucleotide molecule placed at either side entrance of the pore. Overall the 225-ns MD simulations (S1 Table) showed no complete permeation event for either ATP or AMP, whatever the KCl concentration, either 0.1 M or 1 M KCl (S1 Fig.). Sporadic entries of the metabolites into the pore were observed, however, at physiological KCl concentration (0.1 M). In rare cases, the metabolite went as far as the central region of the pore and formed interactions mainly via its phosphate group, with positively charged residues located in the N-terminal helix and on each side of this segment (S2 Fig.). Only one entry of AMP and none of ATP was observed in the trajectories produced at 1 M KCl. The absence of complete crossing of ATP was also noted in previously reported MD simulations of mVDAC1 [34].

**Fig 1:**
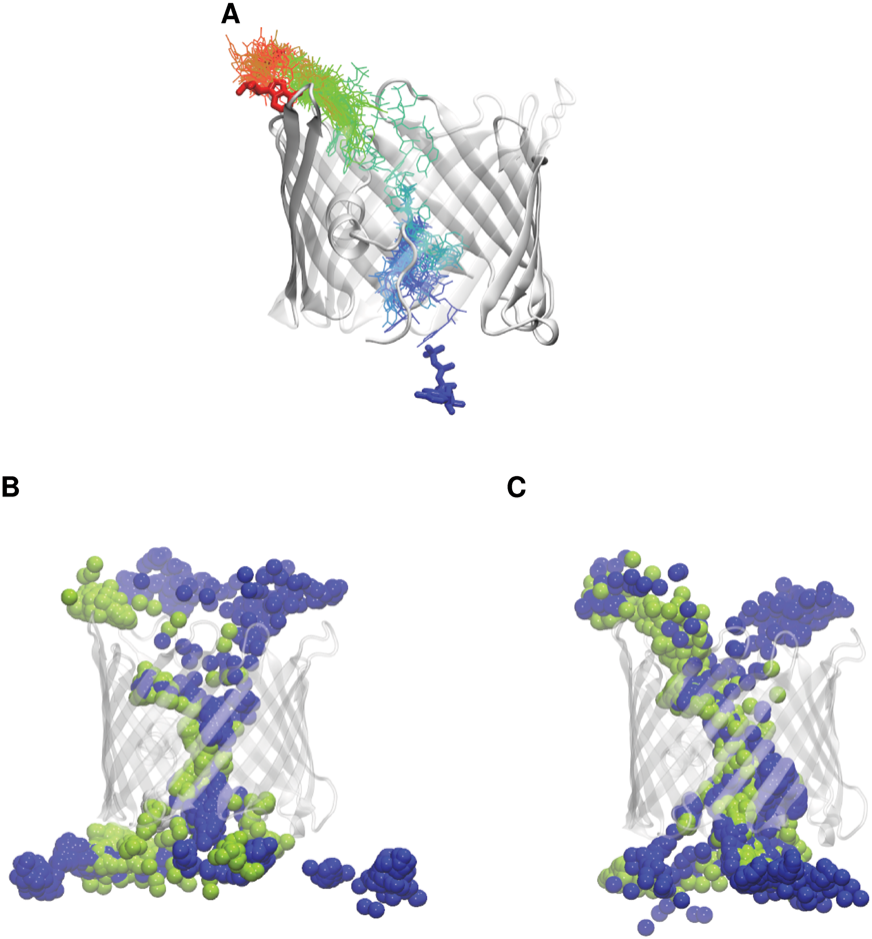
Permeation pathways followed by ATP and AMP. Different views of mVDAC1, extracted from the MD trajectories performed with an imposed transmembrane potential of 500 mV. (A) Translocation path followed by ATP through the pore during one of the observed permeation events. ATP structure is depicted as thicker sticks at the initial (blue) and end (red) positions and as thinner sticks along the trajectory. It is colored according to a scale from blue to red points separated by a 0.1-ns time step. (B, C) Pathways followed through the VDAC pore by (B) AMP and (C) ATP, as shown by the positions of the terminal phosphate groups, depicted as spheres every 0.2 ns along 10 different 50-ns trajectories performed at 0.1 M (green) and 1 M (blue) KCl concentration.

To enhance permeation of these two anionic metabolites through the VDAC pore, a driving force in the form of an electric potential was applied across the membrane in the MD simulations [53–56]. No full permeation event was observed in the overall 290-ns simulations carried out with an applied voltage of 50 mV; S1 Table). With a 500-mV transmembrane potential, several translocation events occurred for ATP and AMP irrespective of the KCl concentration (Fig. 1A, S3 Fig.). The need to increase the applied voltage so as to observe full translocation events can be rationalized by the rather low flow of ATP through VDAC that is one ATP per 100 μs, in the presence of 1 mM ATP and a transmembrane potential of -20 mV [2]. The superposition of all the instantaneous configurations shows that ATP and AMP followed similar and rather specific pathways when crossing the VDAC pore (Fig. 1B, C), in contrast to what was found for the permeation of small inorganic anions [38,40].

We monitored along these trajectories the frequency of finding the terminal phosphate group of the metabolite considered inside the VDAC pore (S4A,B Fig.). At 0.1 M KCl, the region located between 5 and 10 Å in the pore was markedly populated by ATP. This site was also occupied in the AMP simulations, albeit to a lesser extent. A second site more scarcely populated and positioned between -5 and -10 Å occurred for both ATP and AMP. At 1 M KCl, two major sites were also found, located at about the same positions as in the 0.1 M trajectories. They were less populated, however, and about equally occupied. For ATP, the major site occurring at 0.1 M KCl suffered marked depletion at 1 M KCl, whereas the occupancy of the second site, occurring between -5 and -10 Å, extended farther towards the intermembrane entrance.

**Fig 2:**
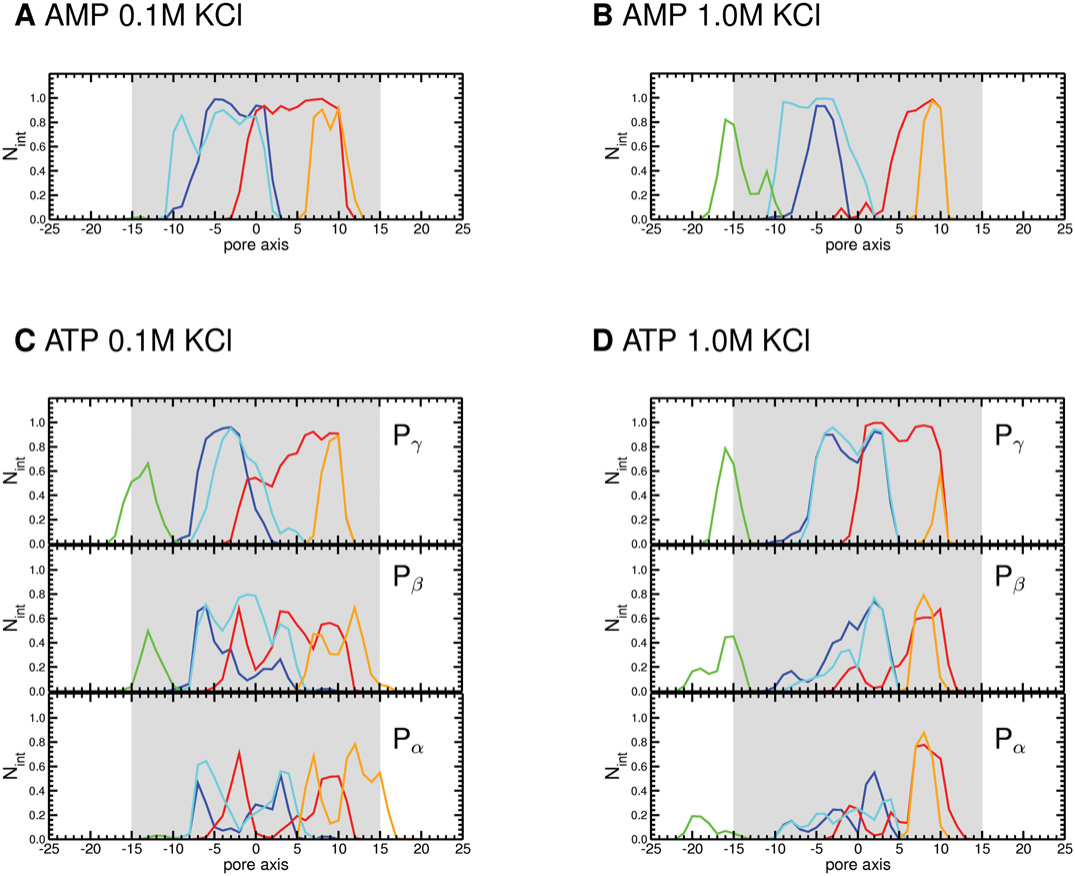
Protein residues interacting with ATP or AMP in the pore. Time-averaged number of interactions (N_int_) of each of the five positively charged residues (K12-blue, R15-red, K20-cyan, K119-green, R218-orange) with (A-B) the phosphate group of AMP and (C-D) the α-, β-, and γ-phosphate groups (P_α_, P_β_, and P_γ_) of ATP along the pore axis as defined by the position of the terminal phosphate in the presence a KCl concentration of either 0.1 M (A,C) or 1 M (B,D).

These trajectories were further monitored to identify the residues most frequently found in the vicinity of each metabolite. In the two major sites populated by ATP and AMP (S4A-B Fig.), two groups of positively charged residues were found to form ionic interactions with the terminal phosphate group of each metabolite (S4C-F Fig.). These two main clusters of basic residues were similar for ATP and AMP, irrespectively of the salt concentration (Fig. 2). The site extending roughly from -5 to 10 Å along the pore axis contained K12, R15, and K20, which all belong to the N-terminal helix. The second site, covering the pore from about 5 to 10 Å, included R15 and R218. The latter residue is located at the cytosolic entrance of the channel in β15. Other basic residues were also found to be involved in ionic interactions with the metabolites, albeit to a lesser degree. In particular, the region towards the intermembrane side, situated between -15 and -10 Å, revealed the engagement of K119 (Fig. 2 and S4 Fig.). Interestingly, each of the basic residues engaged in an interaction with the phosphate, i. e. K12, R15, K20, R218, and to a lesser extent K119, covered a fairly large portion of the whole pore, thanks to their long and flexible side chains (Fig. 2).

In both ATP and AMP trajectories and irrespective of the KCl concentration, interactions were formed only sporadically between purine moieties and pore residues. Similarly, no persisting hydrogen bonds were found between the adenine ring or the ribose moiety and protein residues. Thus, the permeation pathway of these metabolites through VDAC seems to result mainly from electrostatic interactions between their phosphate groups and protein basic residues.

The number of potassium ions pairing with metabolite terminal phosphates along the VDAC pore was also estimated (S5 Fig.). In the 0.1 M KCl simulation, ATP traveled with about 2 potassium ions over practically the whole length of the pore, apart from two zones where this number dropped to about one. Reduced potassium ion pairing correlated with a marked increase in the number of interactions of the phosphate with protein residues in these two regions. One region encompasses the N-terminal helix, where ATP interacted mainly with K12, R15, and K20, and the other is comprised between this helix and the cytosolic entrance of the pore, where ATP interacted both with R15 and R218 (Fig. 2). In contrast to ATP, AMP crossed the VDAC pore almost unaccompanied by potassium ions (S5 Fig.). In the 1 M KCl simulations, the number of potassium ions pairing with ATP or AMP phosphates increased significantly.

We have so far studied the permeation of the free form of ATP through VDAC. As the Mg^2+^-ATP complex is fairly abundant in the cytosol and the mitochondrial matrix [70] we also investigated the permeation of the magnesium bound form. Several full crossing events of the Mg^2+^-ATP form occurred in the 10 individual MD simulations performed with an applied 500-mV potential (S6A Fig.). Mg^2+^ was never observed to dissociate from ATP nor to change its coordination state in the course of the simulations as also previously reported [34]. The superposition of the configurations showed that Mg^2+^-ATP sampled a slightly broader distribution inside the pore relative to the free form (Fig. 1C and S6B Fig.). However the ATP bound form interacts with basic residues identical to those found with the unbound form but a few ones (S4B and S6C Figs.). These latter residues were mentioned as pertaining to low-probability ATP pathways in a previous study [34]. Noteworthy the comparison of our trajectories either with ATP free or ATP bound form indicated that the association with Mg^2+^ did not significantly alter the structural features of ATP permeation. In that respect our data are in agreement with two previously published permeation studies, one performed with the unbound ATP form [46] and the other with the complex ATP-Mg^2+^ form [34]. In view of these findings and of some experimental observations (see Discussion) we have solely examined, in the following, the translocation energetics of the ATP free form.

### The energetics of metabolite translocation

**Fig 3:**
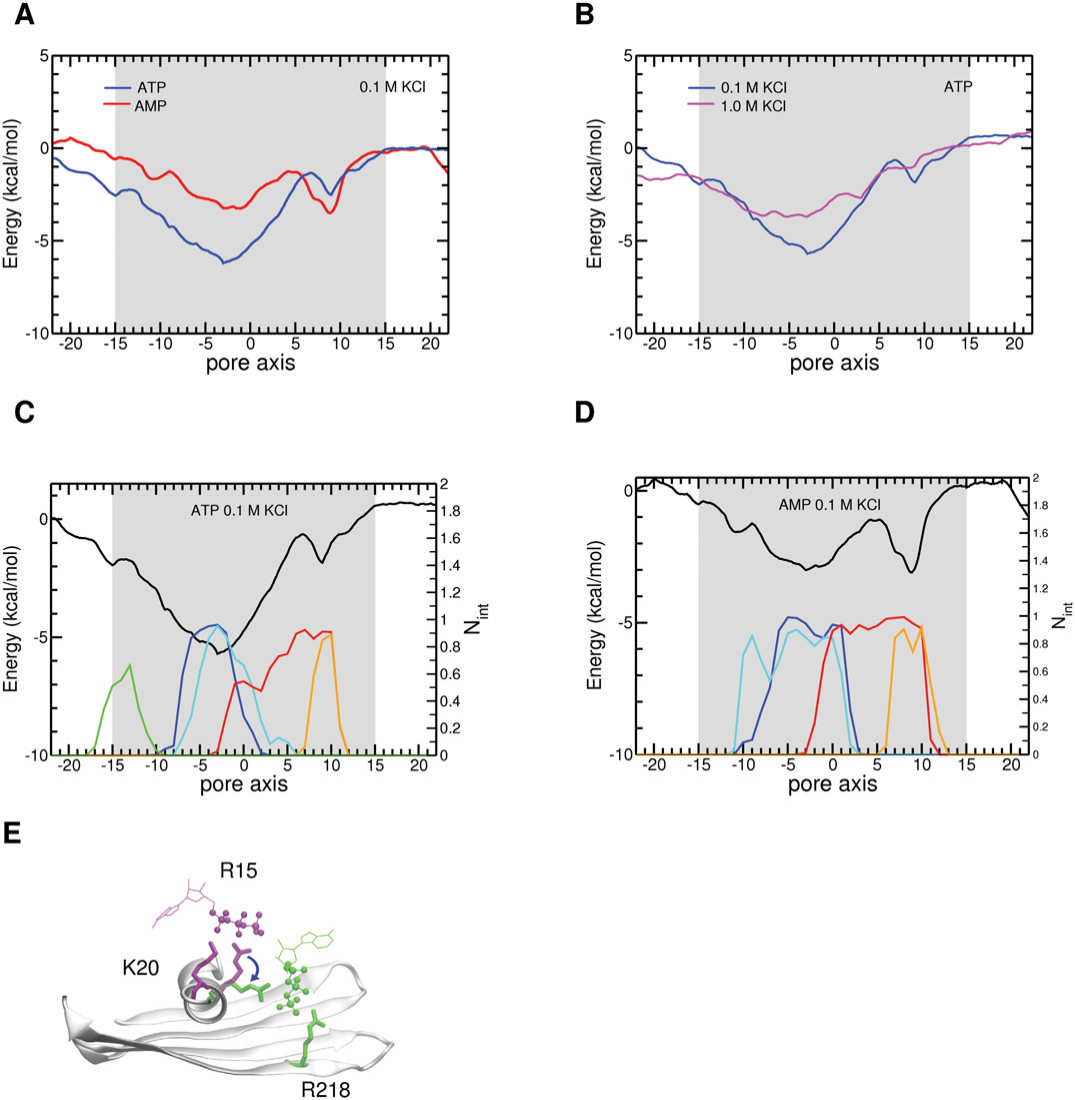
Energetics of ATP and AMP permeation. (A) Permeation free energy profiles of AMP (red) and ATP (blue) in the presence of 0.1 M KCl (B) comparison of the permeation free energy profiles of ATP at 0.1 M (blue) and 1 M KCl (magenta). (C, D) Free energy profiles of ATP and AMP, respectively, at 0.1 M KCl, together with the time-averaged number of interactions (N_int_) formed by their terminal phosphates with VDAC pore basic residues (K12-blue, R15-red, K20-cyan, K119-green, and R218-orange), as extracted from the 500-mV trajectories. Error bars together with the free energy profiles are shown in S7 Fig. (E) View of two consecutive MD conformations (in green and purple) showing ATP migration assisted by basic key residues in its major binding site of the VDAC pore. ATP phosphates are shown as balls and sticks, its sugar and ribose as thin sticks, and the basic protein residues as thick sticks.

Analysis of the MD trajectories indicated that ATP and AMP permeated the VDAC pore in a broadly similar way, apart from the higher number of cations pairing with ATP. To investigate the permeation energetics of these two metabolites, we used ABF [60,61]. The free energy landscape of one single ATP and AMP molecule permeation, obtained using the pore axis as a reaction coordinate, was computed from ~0.9-μs ABF simulations at 0.1 M KCl concentration. The overall shape of the free energy profiles (Fig. 3) was similar for the two metabolites. A deep and broad energy well was found for ATP. This valley corresponded to a site located roughly between -10 and 5 Å and containing the N-terminal helical segment and its cluster of positively charged residues, K12, R15 and K20 (Fig. 3C). The AMP profile featured at the same position a shallower minimum, which might be due to the fact that only a single phosphate group in AMP can form ionic interactions with the positively charged residues of the N-terminus (Fig. 2). The AMP profile featured a second well as deep as its first one. This site corresponded to a region lined by R15 of the N-terminal helix and R218, located close to one mouth of the pore (Fig. 3D). In the ATP profile this minimum was also observed, but it was much shallower than the other well.

Our recent studies have shown that VDAC selectivity towards inorganic ions depends upon the salt concentration [38–40]. This prompted us to investigate the effect of ionic strength on the free energy of ATP permeation. At high KCl concentration, the ATP profile is markedly smoothed out (Fig. 3B). The free energy surface conserves the broad well, albeit with a reduced energy, and does not display the other, shallow energy well. This might be due to screening of the positively charged side chains inside the pore by the increased salt concentration, leading to a reduced probability of ATP binding.

### Mechanisms of phosphate compound permeation through VDAC

In the light of our finding that ATP/AMP permeation characteristics differ from those of chloride, and because of the major engagement of the phosphate groups of AMP and ATP with the basic residues of the VDAC pore during their permeation, we used MD simulations to explore the mechanism of transport of the simplest phosphate anion (P_i_) through VDAC. In this study, the monovalent (H_2_PO_4_^-^) and divalent (HPO_4_^2-^) forms of P_i_ were considered, as they are the most prevalent protonation states around the physiological pH of 7. The molecular system was simulated under a P_i_ salt concentration of 0.2 M to obtain a statistical significance of the calculated properties of the channel [38,40] and also to facilitate the comparison with an experimental study [27]. As we used sodium, in this case, as the P_i_ counterion, we carried out additional MD simulations of NaCl permeation as a control.

**Fig 4:**
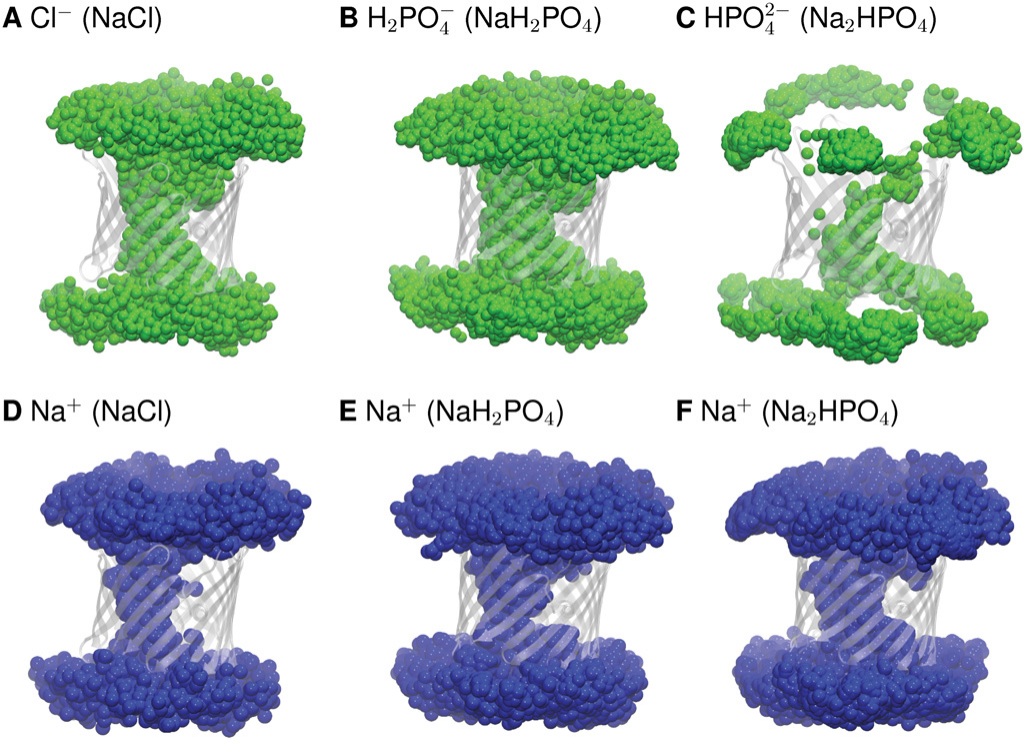
Permeation pathways followed by inorganic ions through the VDAC pore. Cl^-^ (A), H_2_PO_4_^-^ (B), and HPO_4_^2-^ (C) and by Na^+^ in NaCl, NaH_2_PO_4_, and Na_2_HPO_4_ in (D, E, F), respectively. Positions of the anions (green) and Na^+^ (blue) within 10 Å of the protein residues are superimposed, using their positions extracted every 0.2 ns from the 200-ns trajectories of NaCl and NaH_2_PO_4_ and the 300-ns trajectory for Na_2_HPO_4_. The protein is shown as a transparent-white cartoon. For clarity, only the position of the phosphorus atom of each P_i_ ion is depicted.

Analysis of the MD simulations demonstrated the absence of any specific pathway through the pore for the permeation of chloride, and the monovalent form of P_i_ as well as that of the sodium counterion (Fig. 4). This is in line with our previous reports on KCl [38–40]. Also, as previously observed for KCl, the NaCl trajectories featured no long-lived specific (> 5 ns) interactions for either Cl^-^ or Na^+^ (S8 Fig.). In simulations with NaH_2_PO_4,_ the monovalent Pi and Na^+^ formed scattered short-lived interactions though a few long-lived interactions were also observed (S8 Fig.). This thus suggests a lack of defined pathways for the permeation of NaH_2_PO_4_, as observed for sodium and potassium chloride [38].

In clear contrast to chloride and H_2_PO_4_^-^, divalent HPO_4_^2-^ followed a specific pathway (Fig. 4C) and induced long-lived interactions with a few privileged residues of the pore, i. e. K12, R15, K20, K96, K119, and R218, revealing binding sites (S8E Fig.). In these simulations, Na^+^ permeated the pore by forming relatively long-lived protein interactions, but without following any specific pathway (Fig. 4 and S8F Fig.). Na^+^ ions also interacted with several acidic residues, not only loop residues as observed in the simulations with monovalent P_i_ but also residues located inside the pore (S8D, F Fig.). These interactions might favor better translocation of Na^+^ in the divalent P_i_ system.

No ion pairing was observed between Na^+^ and either Cl^-^ or H_2_PO_4_^-^, as previously noted for KCl [40] (S9 Fig.). In contrast, we found between 3 and 5 sodium ions around the divalent P_i_ located in the bulk and at the entrances of the pore. Depletion of the sodium ions around the divalent phosphate occurred inside the pore, where the cations were replaced by about 2 to 3 protein residues (K12, R15, and K20 or R15 and R218; S9C Fig.).

The N_anion_/N_cation_ ratio between the time-averaged number of anions (Cl^-^, H_2_PO_4_^-^ or HPO_4_^2-^) and that of cations (Na^+^) visiting the pore can be interpreted as a measure of the ion preference of VDAC under equilibrium conditions (S10 Fig.). In our simulations, this ratio indicated a preference of VDAC for chloride over sodium (N_Cl_/N_Na_ ≈ 3) and of monovalent P_i_ over sodium (N_H2PO4_/N_Na_ ≈ 3), in agreement with experimental measurements [27,71,72]. In contrast, the N_anion_/N_cation_ ratio computed for HPO_4_^2-^ ((N_HPO4_/N_Na_ ≈ 1) revealed a much lesser preference of VDAC for the anion. This observation also agrees with reported measurements showing practically no selectivity of the VDAC towards the divalent form of P_i_ [27]. More importantly, the VDAC anion preference series deduced from our simulations, NaCl>NaH_2_PO_4_>Na_2_HPO_4_, correlated well with the trend deduced from experimentally measured reversal potential [27].

### Energetics of phosphate permeation through VDAC

**Fig 5:**
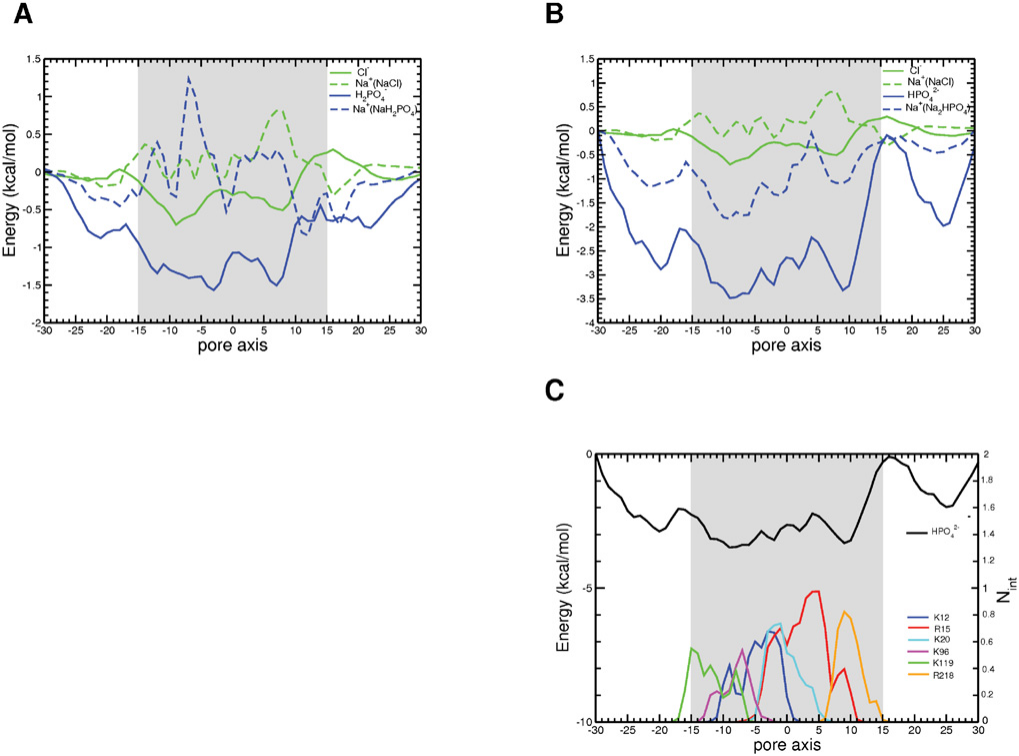
Energetics of inorganic ion translocation through the VDAC pore. Free energy profiles of permeation (A) of Na^+^ and Cl^-^ and of Na^+^ and H_2_PO_4_^-^, and (B) of Na^+^ and Cl^-^ and of Na^+^ and HPO_4_^2-^ (C) of HPO_4_^2-^ together with the time-averaged number of interactions formed by the phosphate with the basic pore residues (K12-blue, R15-red, K20-cyan, K96-magenta, K119-green, and R218-orange) in the pore. Error bars together with the free energy profiles are shown in S11 Fig.

The individual free energy profiles for the transport of Cl^-^, H_2_PO_4_^-^, HPO_4_^2-^, and their Na^+^ counterion in the different simulations are shown in Fig. 5 and S11 Fig. The different anions showed different energy surfaces, but with a common feature: the existence of energetically favorable binding regions. For NaCl, two distinct energy wells were found for the anion, matching a location around the N-terminal helix and a region between this helix and the cytosolic entrance. These two valleys corresponded to the two main barriers in the energy profile of sodium. These profiles suggest that chloride transport is favored over sodium transport, as previously observed for KCl [38].

The VDAC pore attracted more the monovalent form of P_i_ than chloride. The anion profile featured two wells, matching those of chloride but about 3 times as deep. The valley spanning the central region containing the N-terminal helix was also broader. The corresponding profile for Na^+^ was not strongly affected as compared to that observed for NaCl.

The VDAC pore had a profound impact on the free energy surface of the divalent form of the phosphate ion. As for chloride and monovalent P_i_, the free energy surface displayed two valleys, but they appeared much more attractive for H_2_PO_4_^2-^. The broader minimum mapped to K12, R15, and K20, located in the N-terminal segment, and K96 and K119, found in β6 and β7 close to the intermembrane side of VDAC. The other minimum was lined by R15 and R218 (Fig. 5C). Remarkably, the Na^+^ profile was also affected in this case, showing almost no energy barrier for VDAC. This might be due to the presence of a high number of HPO_4_^2-^ in the pore favored by the long-lived interactions formed with protein basic residues (S8E Fig.) thereby screening their positive charges. This may, in turn, make the pore more attractive to cations such as Na^+^ and allowing it to bind to protein residues located inside the pore, such as D9 (S9D Fig.). This absence of an energy barrier may also provide a rationale for the lack of selectivity of VDAC towards the divalent P_i_ (S10 Fig.) [27].

### Effect of ATP and AMP addition on single-channel conductance

On the basis of current-noise measurements performed on fungal VDAC at 1 M salt concentration, it has been suggested that AMP and UTP diffuse freely through the pore, whereas ATP and NADH permeate via specific binding sites [31]. In contrast to these findings, a recent NMR study has evidenced binding of ATP, UTP, and GTP to the same region in the hVDAC1 pore [20]. Our computational data obtained on mVDAC1 at 0.1 M salt concentration suggest that AMP and ATP permeate the VDAC pore through interactions with almost identical protein residues, and feature a similar overall shape of their energy profiles, albeit with different well depths (Fig. 3). We also show major differences in ion pairing for ATP and AMP (S5 Fig.). These observations prompted us to assess the impact of ATP and AMP on the conductance of VDAC.

In the presence of either ATP or AMP, conductance measurements were performed on PcVDAC. This plant channel was indeed shown to feature functional and structural properties similar to those of mammalian VDAC1 [10]. Moreover studies combining molecular simulations and electrophysiological experiments reported similar features regarding the permeation of small inorganic ions [10,39,40]. Lastly, the three basic residues of the central helix identified to form a binding site for ATP, AMP and the divalent P_i_ are strictly conserved between mVDAC1 and PcVDAC. The peripheral residues (K119 and R218 in mVDAC1) are not, however, their role could be taken over by structurally equivalent basic residues as shown by a superposition of a 3D model of PcVDAC [10] and of the mVDAC1 structure (S12 Fig.).

To be able to detect a significant effect on the measured conductance a concentration of metabolite of 25 mM was used [31]. The measurements were performed in both 0.1 and 1 M NaCl. The choice of a reference experiment was complicated in the experiments performed at 0.1 M, because addition of 25 mM AMP or ATP increased the Na^+^ concentration by 50 mM or 100 mM, respectively. We thus used as references two different NaCl solutions: one at 0.1 M, i.e. the same as prior to metabolite addition, and one at 0.15 or 0.2 M, to take into account the effect of adding AMP or ATP, respectively.

**Fig 6:**
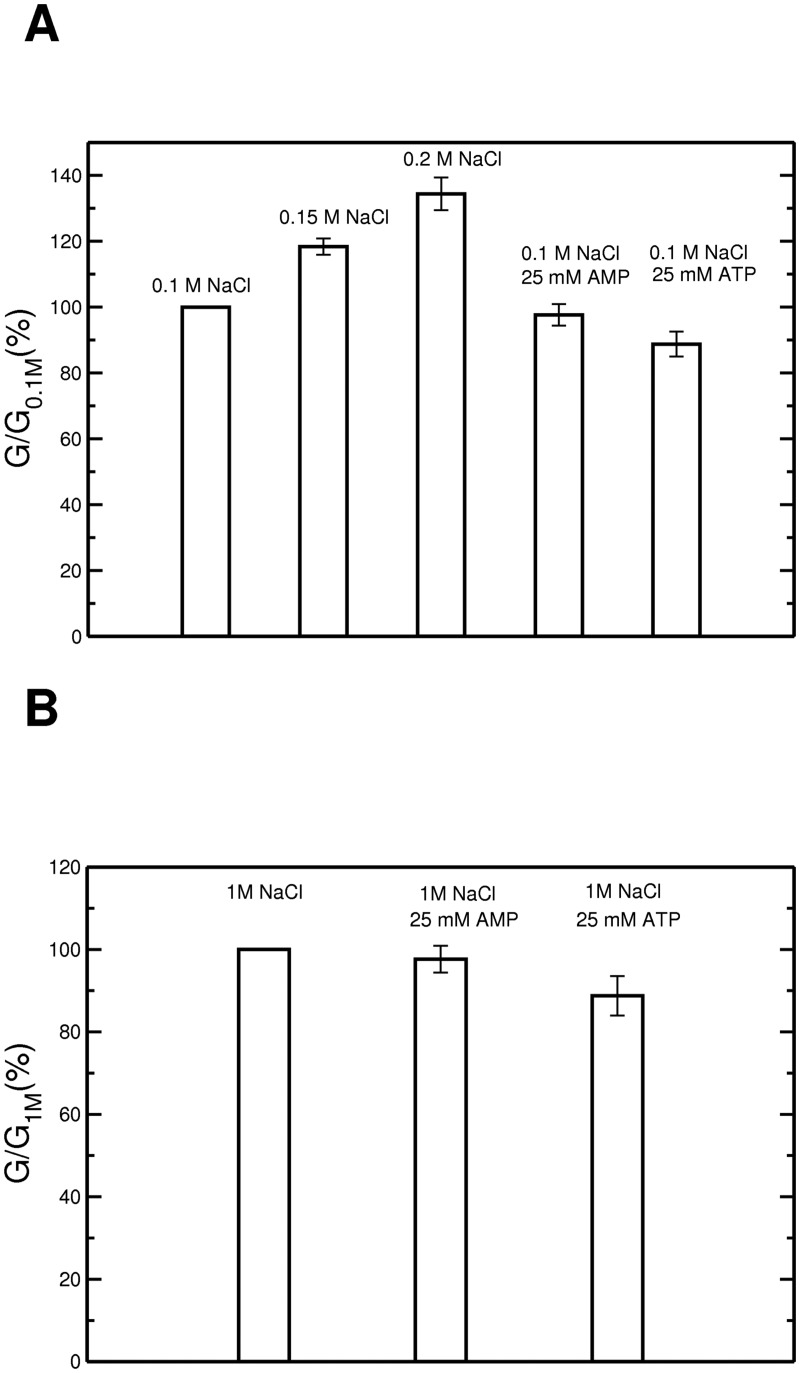
Effect of ATP and AMP on PcVDAC conductance. (A) 0.1 M NaCl or (B) 1 M NaCl in the *trans* compartment. Each *cis* compartment concentration is indicated in the plot. Conductance is normalized with respect to the corresponding value in 0.1 or 1 M NaCl. The metabolite is added to the *cis* compartment, which raises the Na^+^ concentration by about 50 mM or 100 mM in the presence of AMP or ATP, respectively (see text).

At 0.1 M NaCl, addition of ATP induced a decrease in channel conductance, ranging from 10 to 34% depending on the control experiment (0.1 or 0.2 M NaCl) used as a reference (Fig. 6A). Adding AMP either had no effect or caused a change of about 25% in the conductance, according to the control experiment to which the measured values were compared (Fig. 6A). At 1 M NaCl, adding 25 mM AMP did not affect VDAC conductance, while adding ATP decreased its magnitude by about 10% (Fig. 6B) as previously observed on rat VDAC [73].

## Discussion

In our efforts to understand in depth the mechanism of permeation of metabolic compounds through VDAC, we have investigated the transport through this channel of several anions, ranging from small inorganic ions to large metabolites, with a focus on ATP and AMP.

Our computational study shows that VDAC prefers chloride and monovalent phosphate over sodium (S9 Fig.), in agreement with experimental selectivity measurements [27]. We have identified no separate pathways (Fig. 4A, D) and no long-lived protein interactions (S8A, B Fig.) for the flow of chloride and sodium across VDAC, and this corroborates data previously obtained for the permeation of potassium chloride [38–40]. The difference between the height of the energy barriers for sodium ions (~ 1 kcal/mol) and the depth of the energy wells for chloride (~-0.5 kcal/mol) is low (Fig. 5A), in keeping with the moderate selectivity of VDAC for chloride versus sodium [71,72]. The permeation process of H_2_PO_4_^-^ resembles that of Cl^-^, apart from the occurrence of a few more frequent long-lived interactions formed by the phosphates with positively charged residues (S8 Fig.). The potential of mean force of the monovalent form of P_i_ ion also features two minima, mapping to regions of the pore similar to those of chloride but deeper (Fig. 5A). We have previously proposed that the preference of the channel for monovalent anions might stem from the existence of an electrostatic potential generated by a few positively charged residues located mainly in the central region of the pore [38–40]. The fact that we have observed no specific pathways and no binding sites indicates that this mechanism applies to both NaH_2_PO_4_ and NaCl (Fig. 4).

In dramatic contrast to this situation, the divalent anion HPO_4_^2-^ forms long-persisting interactions with a small number of positively charged residues (S8E, F Fig.). Like the energy profiles of chloride and monovalent P_i_, that of HPO_4_^2-^ features two valleys, but they are much more attractive (~-4 kcal/mol; Fig. 5B) than those of the other two anions. The VDAC pore also offers a more favorable environment for the sodium ions of divalent phosphate than for those of the monovalent phosphate H_2_PO_4_^-^, as shown by its energy profile displaying energy minima instead of barriers. In keeping with these observations, the preference of VDAC for anions over Na^+^ was found to plummet in the case of the divalent form of P_i_ (S10 Fig.), in agreement with the experimental data [27]. A specific pathway is clearly apparent for HPO_4_^2-^, in contrast to the other two anions studied here (Fig. 4). Ion pairing also clearly differs for monovalent and divalent Pi (S9 Fig.). The free energy profile for Na^+^ shows no energy barrier in the case of the divalent salt, in contrast to what is observed for the monovalent salt (Fig. 5). This feature may arise from shielding of the positively charged residues in the pore by the phosphate anions, favoring the cation translocation. This provides an interpretation for the measured loss of VDAC anion selectivity in the case of divalent P_i_ [27].

The marked differences observed for monovalent and divalent phosphate permeation cannot be due to a “size exclusion” effect, given the small difference in size between the two phosphate forms. These disparities are more likely to arise from the total charge carried by the two anions and/or its distribution over each anion. The selectivity of VDAC towards different types of small ions thus seems to arise from fine-tuned interactions between both the anions and their counterions with protein residues.

ATP and AMP are anionic metabolites far more complex than the small inorganic ions, chloride and phosphates, we have investigated here. Yet our simulations show that the permeation of these two compounds and of divalent phosphate shows similar features (compare Figs. 1B, C and 3 for ATP and AMP with Figs. 4C, F and 5B, C for divalent P_i_) and remarkably differs from those of monovalent P_i_ and chloride. Interestingly, the AMP energy profile shows two minima (Fig. 3A), featuring well depths of similar magnitude to those found for HPO_4_^2-^ (Fig. 5A) which bears the same total charge as AMP. ATP also shows two minima in its free energy profile (Fig. 3A), corresponding to the same regions of the pore as found for AMP and HPO_4_^2-^. One of the energy valleys maps to the constriction region near the center of the protein, and is lined mainly by a cluster of three positively charged residues of the N-terminal helix K12, R15, and K20 (Fig. 3C, D), whose contribution has been stressed in previous experimental and theoretical studies [18–20,34,46]. The potential involvement of K12, R15, and K20 in the permeation of these compounds is also supported by evolutionary observations, as these three residues are conserved among orthologs of VDAC (Table 1). For ATP, this well features a broad minimum of about -5 kcal/mol, deeper than the corresponding ones for AMP (~ -3 kcal/mol) and HPO_4_^2-^ (~ -4 kcal/mol). This difference in energy could be due to the occurrence of more persistent ionic interactions established by the three ATP phosphates (Fig. 2).

**Table 1:** Conservation across VDAC species of residues interacting with the phosphate ions and anionic metabolites.

Residue	Secondary structure	Pore Location	ATP/AMP trajectories				0.2 M NaH_2_PO_4_/Na_2_HPO_4_ trajectories		Conservation		Exp. ref.	Theo.ref
			AMP		ATP		H_2_PO_4_^-^	H_2_PO_4_^2-^	Cons [%]	Changed to K/R [%]		
			0.1 M	1.0 M	0.1 M	1.0 M						
K12	α-helix	CEN	✓	✓	✓	✓		✓	92.8	R 6.5	[18,20,34]	[34,46]
R15	α-helix	CEN	✓	✓	✓	✓	✓	✓	51.1	K 24.5	[18,20]	[34,46]
K20	α-helix	CEN	✓	✓	✓	✓	✓	✓	79.1	R 16.5	[18–20,34]	[34,46]
K32	β1	IS				✓			56.8	R 3.6		
K34	β1-β2 loop	IS	✓		✓				50.4	R 0.7	[20]	
K53	β3	CYT		✓		✓			46.8			[34,46]
K61	β3	CEN			✓	✓			63.3	R 2.9	[20]	
R63	β3	IS				✓			7.2	K 64.7		[34]
R93	β5-β6 loop	IS		✓		✓			7.9	K 31.7	[20]	[34]
K96	β6	IS						✓				
K115	β7	CEN		✓			✓		43.9		[18]	[34]
K119	β7	IS		✓	✓	✓		✓	45.3	R 7.2	[18,20]	
R139	β9	CEN		✓					7.2			[34]
K200	β13-β14 loop	IS			✓				8.6	R 2.2		
R218	β15	CYT	✓	✓	✓	✓	✓	✓	35.5	K 4.3	[20]	[34]
K224	β15	CEN		✓					28.8	R 3.6		
K266	β18-β19 loop	CYT			✓				30.2	R 2.2		

Sequence conservation of residues identified as interacting, in the MD simulations, with the terminal phosphate of AMP or ATP, H_2_PO_4_^-^, or HPO_4_^2-^. The other columns list, respectively, the secondary structure type, location in the pore, and the experimental and theoretical study reference reporting the importance of this residue for ATP binding (last two columns). Residue conservation was determined using a previously constructed multiple sequence alignment including 139 VDAC sequences [10]. Abbreviations used: CEN, central region of the pore, CYT, cytosolic side, IS, intermembrane space side.

The second well maps to a binding site located towards the cytosolic side of the protein and encompasses R15 and R218 (Fig. 3C, D). At this location the phosphate of ATP or AMP interacts persistently with these two residues in a configuration where the metabolite is caught in a sort of pincer movement between R15 and R218 (Fig. 3C, D). Interestingly, R218 has been found in a recent NMR study to interact with ATP [20] and its role in ATP permeation has also recently been reported in a combined experimental and computational study [34]. This second site however differs from that identified in an umbrella-sampling MD study [46]. The discrepancy might be due to different simulation methodologies and/or simulation times, which can impact the energy landscape and shallow minima in particular. Remarkably, the pathway followed by ATP in our simulations is similar to the most probable path revealed in a Markov state Model study [34].

Our simulations also show that ion pairing is remarkably different for ATP and AMP (S5 Fig.). AMP travels almost freely in the VDAC pore, as do Cl^-^ and H_2_PO_4_^-^ (S9 Fig.). In contrast, ATP and HPO_4_^2-^ are accompanied by several sodium ions, except in the energy minimum regions, where they are stripped of their counterions to form strong interactions with positively charged residues. Similar ion pairing of ATP and of the divalent form of P_i_ cannot be explained on the basis of charge, as they differ by 2 full charges. In the case of HPO_4_^2-^, the total charge of the anion and the high accessibility of three of its oxygens might plausibly explain the fairly large number of sodium ions pairing with it. As for ATP, its capacity to complex the cation might be enhanced both by its charge and by its three phosphates and their conformation.

ATP is known to strongly chelate magnesium in solution [74] and the ATP-Mg^2+^ complex is undoubtedly the most abundant form of ATP in the cytosol and the mitochondrial matrix [70]. These observations point to the bound ATP as being potentially the major species permeating VDAC. This is far from obvious however as it was shown that only the free form of ATP is transported through the inner mitochondrial membrane by the ATP/ADP carrier [75] and that the fully charged form of ATP can permeate VDAC *in-vitro* [2,29]. That the reduced ATP binding affinity to VDAC upon Mg^2+^ complexation [2,18,20,29] was interpreted differently [18,20,34] makes the issue even more complex. However, the comparison of our simulations (S6 Fig.) with the free and Mg^2+^-bound ATP, suggests that the chelation does significantly alter neither the pathways followed by the metabolite through the channel nor its interactions formed with basic residues.

VDAC is remarkable in that it can discriminate between anions, using the distribution of lysines and arginines in its pore (Figs. 2 and 3). One cluster of basic residues, located in the N-terminal helix, acts as a major selectivity filter for both smaller and larger ions. Other isolated positively charged residues, located at each mouth of the pore, facilitate the entrance of incoming divalent phosphate, AMP, and ATP. Regulation of ion permeation by VDAC occurs at different levels. First, VDAC differentiates on the basis of the total electric charge carried by the anion, as demonstrated by the dramatic differences observed between the permeation characteristics of HPO_4_^2-^ on the one hand, chloride and H_2_PO_4_^-^ on the other, and also by the similarities between HPO_4_^2-^ and AMP permeation. In addition, VDAC also appears sensitive to the number of phosphates contained in the anion, as suggested by the observed differences in the energy profile of ATP versus those of AMP and HPO_4_^2-^.

At high salt concentration, the conductance of PcVDAC decreases by 11% in the presence of ATP, whereas AMP has no impact (Fig. 6). This is in line with previous observations on rat [73] and fungal VDAC [31]. In a 0.1 M salt solution adding ATP reduces PcVDAC conductance by 11 to 35% while the addition of AMP decreases it by 2 to 25% (Fig. 6). This reduction in conductance might be due to entry of the metabolite into the channel, hindering the permeation of small inorganic ions.

Our simulation results provide a rational basis for these permeation features. First, the differences observed in the free energy profiles of ATP in 1 M versus 0.1 M salt, i. e. a more shallow energy well characterizing the major minimum, are consistent with a lesser impact of ATP on the VDAC conductance at 1 M. Similarly, the observation of a much weaker effect of AMP than of ATP on the conductance of the channel at 0.1 M might be rationalized on the basis of their energy profiles showing that AMP permeation is marked by two energy wells of about equal depth, whereas the ATP energy profile features one broad and deeper valley. These energetic characteristics support the occurrence of sites in the channel more favorable to dwelling of ATP molecules, and hence to an enhanced probability of ATP binding to specific residues, in particular in the N-terminal helix region. These events should cause a reduction in the flow of small ions across the pore. Furthermore, binding of this anionic metabolite to positively charged residues is likely to diminish the electrostatic attraction of anions inside the pore (the so-called Donnan effect), which in turn should alter the VDAC conductance. The fairly good agreement between the experimental data measured on rat [73], fungus [31] and plant VDAC (Fig. 6) and our simulation data obtained on mVDAC1 is not surprising as these VDAC feature similar electrophysiological and structural properties typical of the canonical VDAC [10,39]. Moreover, important residues identified in this study are mostly conserved throughout VDACs of the different eukaryotic organisms (Table 1).

**Fig 7:**
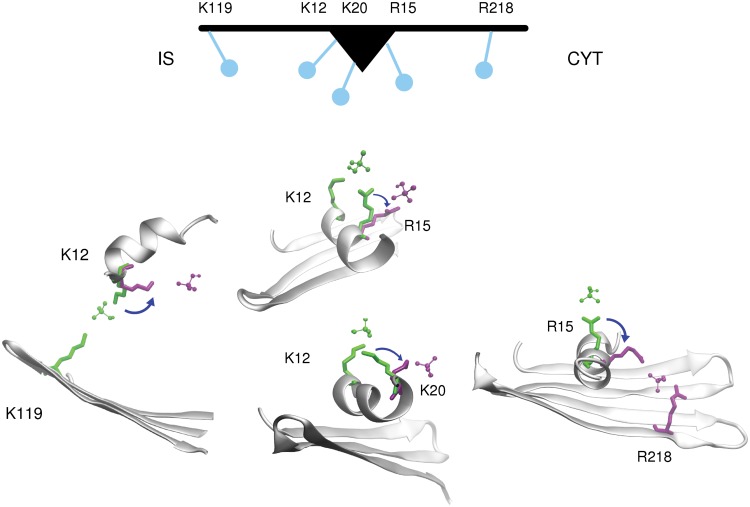
The charged brush mechanism of VDAC. Schematic representation of the positively charged key residues acting as a charged brush to facilitate permeation of the divalent form of P_i_, AMP, and ATP along the VDAC pore. Below are represented successive MD conformations (in green and purple) showing these positively charged side chains undergoing a sweeping motion assisting the translocation of P_i_ along the pore. P_i_ are shown as balls and sticks, and the basic residues as thick sticks. CYT and IS stand for the cytosolic and the intermembrane side, respectively.

The VDAC pore is lined with positive residue side chains notable for their flexibility. Altogether, residues K12, R15, and K20 in the central region of the pore and R218 and K119 on the cytosolic and intermembrane sides, respectively, take turns to get ATP, AMP, and divalent phosphate moving along the whole length of the VDAC pore. Their long and flexible side chains, together with their particular locations in the pore enable them to accompany the metabolite along the pore, facilitating the passage of anionic molecules (Fig. 7). This mechanistic view is reminiscent of the “charged brush” concept proposed in a study of phosphate transport through the bacterial outer membrane protein OprP [76], proposed to behave as a brush-like nanopore [77]. Interestingly, all the basic residues forming the “charged brush” have been reported to affect ATP binding to VDAC in experimental studies [18,20].

Even though the ability of VDAC to differentiate between different anions is globally of electrostatic origin, its pore displays clearly different mechanistic features inducing nontrivial disparities in the translocation of charged species. An in-depth understanding of the mechanism of ion selectivity in a wide nanometric aqueous pore such as the VDAC pore could provide a better grasp of the permeation mechanisms of other biological β-barrel channels. VDAC, a eukaryotic pore, should contribute to addressing general questions about electrostatic interactions in the confinement of an aqueous molecular pore.

## Supporting Information

S1 Fig Initial positions of the ATP in the "classical" and ABF MD simulations. (A) In the MD simulations five randomly chosen initial positions of ATP molecules in the intermembrane space side of the pore are depicted. The protein termini are located in the inner membrane space side [52]. (B) All the initial positions of ATP in each window of the 0.1 M KCl ABF MD simulation are depicted in the VDAC pore (left). To highlight their conformational internal variability these positions overlaid using their phosphate groups are also shown (right). The colors for ATP are chosen randomly. The figures were prepared with vmd [66].
(EPS)

S2 Fig Metabolite permeation in the absence of a transmembrane potential. Time-dependent position of (A) AMP and (B) ATP in MD simulations performed in absence of a transmembrane potential at 0.1 M KCl starting from 5 different randomly chosen positions in either the cytosolic (cyt; on the top) or the intermembrane space (IS; on the bottom). The simulation 1, 2, 3, 4 and 5 are depicted in yellow, red, magenta, blue and green respectively. Percentage of interactions formed between the terminal phosphate group of (C) AMP or (D) ATP and positively charged residues of mVDAC1 along the 0-mV trajectories colored following the scale.
(EPS)

S3 Fig Metabolite permeation in the presence of a transmembrane potential. Time-dependent position of (A, B) ATP and (C, D) AMP along the 500-mV trajectories at (A, C) 0.1 M and (B, D) 1 M KCl. The simulations 1, 2, 3, 4 and 5 started from 5 different randomly chosen positions of ATP and AMP placed either in the cytosol (cyt; on the top or the intermembrane space (IS; on the bottom) are depicted in yellow, red, magenta, blue and green respectively.
(EPS)

S4 Fig Locations of the phosphate group of ATP or AMP and its interactions with VDAC residues. (A, B) Relative number of MD snapshots featuring the terminal phosphate of AMP and ATP along the position z of the simulation box at (A) 0.1 M and (B) 1 M KCl. The grey stripe represents the mVDAC1 pore width. Percentage of interactions formed between the terminal phosphate group of (C, D) ATP and of (E, F) AMP with positively charged residues either at (C, E) 0.1 M and (D, F) 1 M KCl colored according to the scale shown on the right side. Data were computed from the 500 ns trajectories performed with a 500-mV transmembrane potential.
(EPS)

S5 Fig Ion-pairing with ATP and AMP. Time averaged number of potassium ions in close vicinity to ATP or AMP terminal phosphate (purple) computed from the 500-mV trajectories at 0.1 M and 1.0 M KCl. Time averaged number of interactions between all protein residues and the phosphate groups are shown in black.
(EPS)

S6 Fig Mg^2+^-ATP permeation at 0.1 M KCl in the presence of a 500-mV potential. Time-dependent position of (A) Mg^2+^ complexed ATP along the trajectories starting from 5 different randomly chosen positions of Mg^2+^-ATP in either the cytosolic (cyt; on the top) or the intermembrane space (IS; on the bottom). The simulation 1, 2, 3, 4 and 5 are depicted in yellow, red, magenta, blue and green respectively. (B) The translocation pathways followed by Mg^2+^-ATP through the pore, as shown by the positions of the terminal phosphate group, depicted as blue spheres every 0.2 ns along 10 different 50-ns trajectories. (C) Percentage of interactions formed between the terminal phosphate group of ATP with positively charged residues colored according to the scale shown on the right side.
(EPS)

S7 Fig Free energy profiles of metabolite permeation and their error estimation. ATP in (A) 0.1 M and (B) 1.0 M KCl and (C) AMP in 0.1 M KCl. Error bars indicate one SE of the data as obtained from 10-ns ABF simulations per window (see Material and Methods).
(EPS)

S8 Fig Interactions of the permeating ions with VDAC as a function of the simulation time. For (A) Cl^-^ and (B) Na^+^ in NaCl (C) H_2_PO_4_^-^ and (D) Na^+^ in NaH_2_PO_4_, (E) HPO_4_^2-^ and (F) Na^+^ in Na_2_HPO_4_. Each formed protein-ion interaction is depicted by a tick along the total of 200-ns trajectories in (A), (B), (C), (D) and of the 300-ns trajectories in (E), (F). Individual trajectories are highlighted through alternating background colors: (A-D) trajectory 1 and 2 white and gray background, respectively; (E,F) trajectories 1, 2, 3 and 4 white, gray, white and gray background, respectively.
(EPS)

S9 Fig Ion-pairing of the different anions. Time averaged number of sodium ions (green) in close vicinity to (A) Cl^-^, (B) H_2_PO_4_^-^ computed from the 200-ns trajectories, and (C) HPO_4_^2-^ computed from the 300-ns trajectories. The time averaged number of interactions between all protein residues and the phosphate group are also shown in red. (D) Time averaged number of interactions formed by sodium ions with protein pore residues (D9-red, E88-green, E203-blue and Y195-magenta), mostly acidic, shown together with the free energy profile of permeation of HPO_4_^2-^ (black).
(EPS)

S10 Fig Ion distribution inside mVDAC1. The ratio N_anion_/N_cation_ computed from the trajectories of KCl [38], NaCl, NaH_2_PO_4,_ and Na_2_HPO_4_ shown as crosses and circles.
(EPS)

S11 Fig Free energy profiles of permeation and their error estimation. (A) Na^+^ and Cl^-^ in NaCl and (B) Na^+^ and H_2_PO_4_^-^ in NaH_2_PO_4_ and (C) Na^+^ and HPO_4_^2-^ in Na_2_HPO_4._ Error bars indicate one SE of the data as obtained from averaging over three blocks of 67 ns for NaCl, NaH_2_PO_4_ and of 100 ns for Na_2_HPO_4_^-^ respectively.
(EPS)

S12 Fig Structurally equivalent basic residues in PcVDAC shown to be involved in metabolite and HPO42- permeation in mVDAC1. Superposition of mVDAC1 structure (red) [5] and a built 3D model of PcVDAC (cyan) [10] reveals the structurally equivalent residues of K12, R15, K20, K119 and R218 of mVDAC1 to K13, R16, K21 (that are also conserved in the sequence) and K90, R205 of PcVDAC. The basic residues are shown as thick sticks colored by atom type and in red for PcVDAC and mVDAC1 respectively. The protein is depicted as cartoon.
(EPS)

S1 Table Overview of the MD simulations of the metabolite permeation. (DOC)
