# Supplementary material for: Dual Mechanism of Ion Permeation through VDAC Revealed with Inorganic Phosphate Ions and Phosphate Metabolites
Source: PLoS One. 2015 Apr 10;10(4):e0121746. doi: 10.1371/journal.pone.0121746 (PMC4393092; doi:10.1371/journal.pone.0121746)
Supplement: S1 Table — (DOC) [file pone.0121746.s013.doc]

| Metabolite | [KCl]  [M] | Trans. Pot. [mV] | Simulation time [ns] for the individual systems corresponding each to a different initial position of the metabolite | | | | | | | | | | Total simulation time [ns] |
| --- | --- | --- | --- | --- | --- | --- | --- | --- | --- | --- | --- | --- | --- |
|  |  |  | Cytosolic | | | | | Intermembrane | | | | |  |
|  |  |  | 1 | 2 | 3 | 4 | 5 | 1 | 2 | 3 | 4 | 5 |  |
| ATP | 0.1 | 0 | 30 | 10 | 15 | 10 | 10 | 10 | 10 | 10 | 10 | 10 | 125 |
|  |  | 50 | 40 | 80 | - | - | - | 100 | - | - | - | - | 220 |
|  |  | 500 | 50 | 50 | 50 | 50 | 50 | 50 | 50 | 50 | 50 | 50 | 500 |
|  | 1 | 0 | 10 | 10 | 10 | 10 | 10 | 10 | 10 | 10 | 10 | 10 | 100 |
|  |  | 500 | 50 | 50 | 50 | 50 | 50 | 50 | 50 | 50 | 50 | 50 | 500 |
| AMP | 0.1 | 0 | 10 | 10 | 10 | 10 | 10 | 10 | 10 | 10 | 10 | 10 | 100 |
|  |  | 50 | 20 | 20 | - | - | - | 30 | - | - | - | - | 70 |
|  |  | 500 | 50 | 50 | 50 | 50 | 50 | 50 | 50 | 50 | 50 | 50 | 500 |
|  | 1 | 0 | 10 | 10 | 10 | 10 | 10 | 10 | 10 | 10 | 10 | 10 | 100 |
|  |  | 500 | 50 | 50 | 50 | 50 | 50 | 50 | 50 | 50 | 50 | 50 | 500 |
